# Quality of Life in Young Adults With Cerebral Palsy: A Longitudinal Analysis of the SPARCLE Study

**DOI:** 10.3389/fneur.2021.733978

**Published:** 2021-11-01

**Authors:** Nicolas Vidart d'Egurbide Bagazgoïtia, Virginie Ehlinger, Carine Duffaut, Jérôme Fauconnier, Silke Schmidt-Schuchert, Ute Thyen, Kate Himmelmann, Marco Marcelli, Catherine Arnaud

**Affiliations:** ^1^UMR 1295 CERPOP, Inserm, Toulouse University III Paul Sabatier, Team SPHERE, Toulouse, France; ^2^Laboratory TIMC-IMAG, Grenoble Alpes University, Department UFR Medicine, Grenoble, France; ^3^Department Health and Prevention, Institute of Psychology, University of Greifswald, Greifswald, Germany; ^4^Klinik für Kinder und Jugendmedizin, Universität zu Lübeck, Lübeck, Germany; ^5^Department of Pediatrics, Sahlgrenska Academy, University of Gothenburg, Gothenburg, Sweden; ^6^Azienda Sanitaria Locale Viterbo, Child and Adolescent Neuropsychiatric Unit—Adult Disability Unit, Viterbo, Italy; ^7^Clinical Epidemiology Unit, University Hospital, Toulouse, France

**Keywords:** cerebral palsy, quality of life, trajectories, impairments, pain, adults

## Abstract

**Introduction:** While most people with cerebral palsy (CP) will have a life expectancy similar to that of the general population, international research has primarily focused on childhood and adolescence; and knowledge about the quality of life (QoL) of young adults with CP, its trajectories, and associated factors remains scarce.

**Methods:** This longitudinal study included young adults with CP living in five European regions and who had previously participated in the SPARCLE cohort as children and/or adolescents. Their QoL in the psychological well-being and social relationships domains was estimated using age-appropriate validated instruments (KIDSCREEN-52 in childhood/adolescence and WHOQOL-Bref in young adulthood). We used generalized linear mixed-effect models with random intercept to estimate long-term trajectories of QoL in both domains and to investigate whether severity of impairment, pain, and seizure influenced these trajectories. We sought to identify potentially different trajectories of QoL from childhood to adulthood using a shape-based clustering method.

**Results:** In total, 164 young adults with CP aged 22–27 years participated in the study. Psychological well-being linearly decreased by 0.78 points (scale 0–100) per year (95% confidence interval (CI) −0.99 to −0.56) from childhood to young adulthood, whereas QoL in the social relationships domain increased (β coefficient 1.24, 95% CI 0.92–1.55). Severity of impairment was associated with reduced QoL in all life periods of the study (childhood, adolescence, and young adulthood): motor impairment with social relationships, and more nuancedly intellectual disability with psychological well-being and social relationships. At all periods, frequent pain significantly reduced psychological well-being, and seizures were associated with lower QoL in the social relationships domain. In both domains, we identified a group of individuals with CP who presented a reverse trajectory compared with the general QoL trajectory.

**Conclusion:** Identification of QoL trajectories and their associated factors yields improved knowledge about the experience of individuals with CP until young adulthood. Further studies are needed to better understand the determinants that have the greatest influence on the different shapes of long-term trajectories of QoL.

## Introduction

It is now recognized that most people with cerebral palsy (CP) enjoy a life expectancy similar to that of the general population (1). This opens new perspectives for understanding of the impact of childhood disability on young adults. Quality of life (QoL) has been considered a key concept in disability research for the past two decades (2). However, its conceptual definition and its measurement in people with disabilities or chronic conditions are complex and still debated in the literature (3). In short, while health-related QoL (HRQoL) refers to those aspects of life that are directly influenced by disability, health problems, or treatments (4, 5), QoL is viewed as a broader concept that is not limited to functioning but encompasses subjective well-being and life satisfaction (6) in line with the approach proposed by the World Health Organization (WHO). Thus, QoL is a multidimensional construct defined as “the individual's perception of his or her position in life in the context of the culture and value system in which he or she lives, and in relation to his or her goals, expectations, norms, and concerns” (7).

Two points deserve particular attention when exploring trajectories of QoL from childhood to adulthood in people with childhood-onset disabilities. First, QoL explores subjective well-being across a range of domains that vary in relevance over time, necessitating the use of age-appropriate instruments. Second, because QoL measurement is weighted by the respondent's internal norms and values, having functional limitations does not systematically mean poor QoL for a given individual (6, 8). Previous studies have nonetheless shown that people with disabilities or chronic conditions report lower QoL scores as a group than the general population. However, the difference varies in magnitude depending on the context, suggesting that these differences may be more related to personal and environmental factors (personal resources, mental health, social support, and socioeconomic status) than to disability (9).

There has been little research on QoL in young adults with CP and even less on change in QoL from childhood. From studies carried out in children and adolescents, we know that pain is a factor strongly associated with a reduced QoL and that severity of impairment and associated conditions also negatively influence QoL (10–15). Deterioration in mobility, high rates of pain, and mental health problems have been observed in young adults with CP (16–20) and justify study of the impact of functional deterioration on QoL in a changing context for these persons who have new expectations and concerns (such as living independently or having romantic and intimate relationships). In addition, from a clinical perspective, it would be interesting to better understand the impact of disability on change in QoL across the life span and to identify what early factors, if any, can limit the deterioration of QoL. To the best of our knowledge, only one Dutch study (21) has explored QoL trajectories from childhood to transition to adulthood in individuals with CP, but it had several limitations. One of these was that it did not include young adults with intellectual disabilities. Overall, it is a major limitation of the existing literature that individuals with the most severe phenotypes are often excluded, notably because of their inability to self-report their QoL. Many authors have raised concerns about proxy-patient reporting, such as its lack of reliability and accuracy, particularly when exploring social or emotional domains (22–24). This leads to a poor representation of this population in studies.

The SPARCLE study aims to document the impact of personal and environmental factors on QoL, with the explicit goal of allowing all individuals with CP to contribute regardless of their severity profile. In this analysis, using longitudinal data from the European SPARCLE cohort, we sought to identify how the QoL of individuals with CP evolves from childhood to young adulthood and whether the severity of impairment and frequency of pain and seizures affect their QoL. We also aimed to determine whether different shapes of QoL trajectories exist in order to better understand the impact of impairment and comorbidities on QoL.

## Methods

### Study Design and Population

The SPARCLE cohort is a multicenter European observational population-based study designed to investigate the role of a comprehensive set of environmental factors and the contribution of health care to social participation and QoL in individuals with CP. The eligible population consisted of individuals born between 1991 and 1997, with a diagnosis of CP according to the Surveillance of Cerebral Palsy in Europe network (SCPE) definition (25). This cohort followed up individuals with CP in three targeted life periods, namely, childhood, adolescence, and young adulthood. The study design has previously been described in detail elsewhere (26).

Initially, the SPARCLE cohort randomly sampled children with CP (aged 8–12 years in 2004–2005, SPARCLE1) from population-based registers in eight European regions with an overrepresentation of the most severe cases and from several independent sources in an additional region. These individuals were followed up when they were adolescents (aged 13–17 years in 2009–2010, SPARCLE2), while an additional sample was recruited in order to maintain statistical power for the third wave of follow-up in young adulthood. The third wave included participants with CP (aged 22–27 years in 2018–2020, SPARCLE3) and was implemented in five of the nine European regions initially involved, namely, southwest and southeast France (departments of Haute-Garonne and Isère), northwest Germany, western Sweden (Göteborg region), and central Italy (Viterbo area).

**Figure 1:**
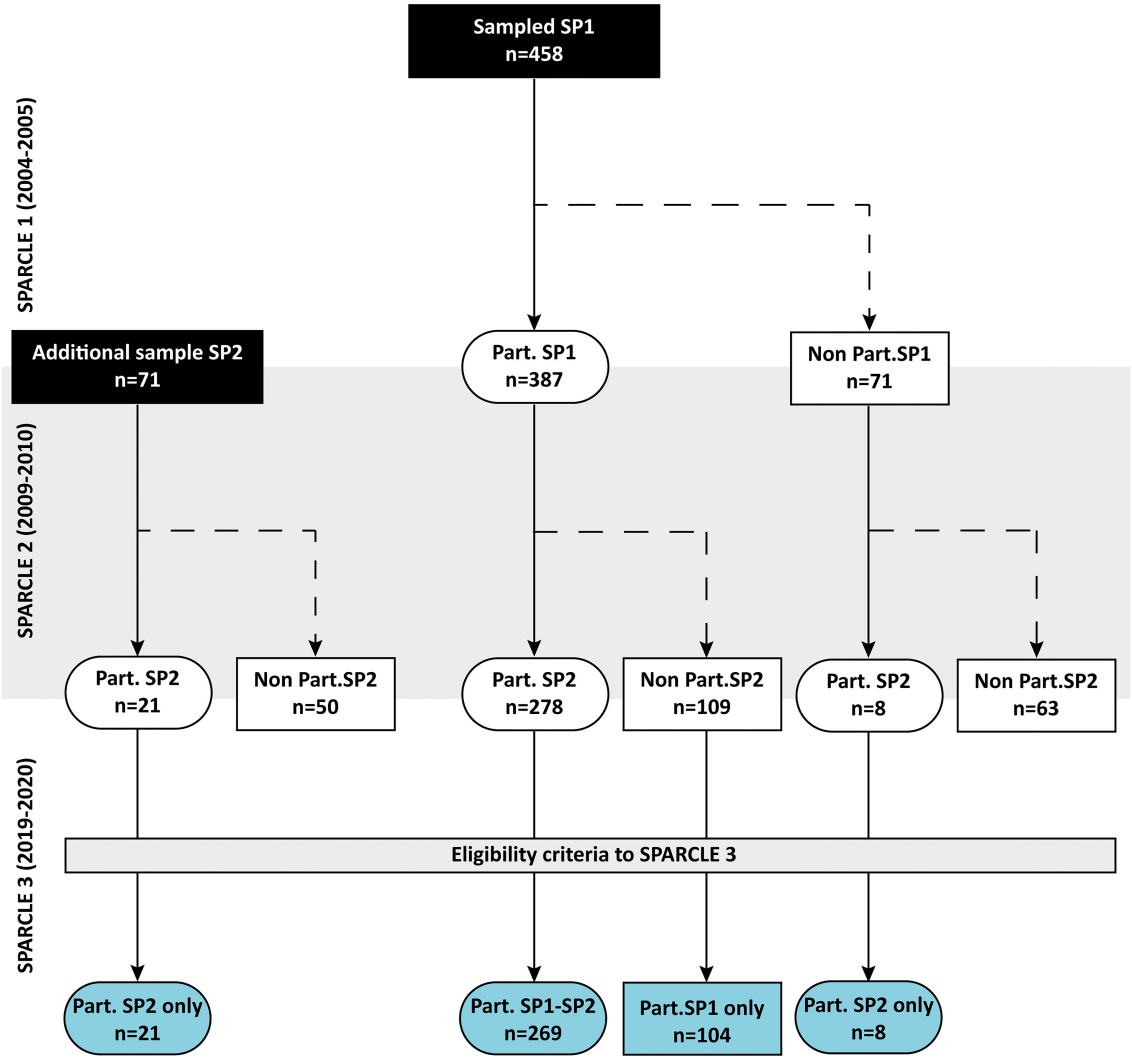
Recruitment process and participation of individuals with CP—SPARCLE cohort—France, Germany, Italy, Sweden. Part, Participants; SP, SPARCLE.

In the above regions, 387 children were enrolled during the first wave. Among them, 278 (72%) agreed to participate in SPARCLE2, and a supplementary sample of 29 adolescents was recruited. At that stage, all participants gave their permission for further contact. Of the 416 individuals with CP who had previously taken part in at least one of the first two waves of the SPARCLE cohort, 14 were excluded because their date of birth did not fit the eligibility criteria. Thus, 402 individuals with CP who had previously participated at least once in the SPARCLE cohort were eligible for SPARCLE3 (Figure 1).

### Dropout

The SPARCLE cohort has been prone to non-response (*n* = 226) due to non-traceability, refusal to participate, or death. To identify factors potentially associated with dropout, we tested separately the following variables collected at inclusion using unconditional logistic regressions adjusted for gender and region: severity of gross motor function impairment [measured with the Gross Motor Function Classification System (GMFCS) (27)], cognitive functioning [estimation of the intelligence quotient (IQ)], parental characteristics [parental educational qualification and parenting stress index (28)], and family structure (combining parental marital status and lifestyle). In addition, we searched for potential interactions with region. We observed a significant increase in dropout rate among individuals with at least one parent who did not complete secondary education (37.6% in individuals who dropped out of the study and 25.0% in participants, odds ratio (OR) 4.0, 95% confidence interval (CI) 1.9–8.4 (Supplementary Material 1).

### Data Collection

In all waves of the SPARCLE follow-up, trained research associates conducted standardized home interviews under identical conditions in each region of the study. Whenever possible, the questionnaire was self-completed, with the interviewer's assistance if necessary. In cases where self-report was not possible, even with help, the interview was conducted with an individual (parent, personal assistant, and partner) who knew the person with CP very well (i.e., they were significantly involved in their daily life).

As in previous waves, the questionnaire used in SPARCLE3 was administered with a logical flow and in a fixed order and elicited information on sociodemographics, physical impairment, comorbidities associated with CP, personal medical history, QoL, social participation, and environment (26). With regard to QoL, as context and expectations evolve along the life span, measurements were carried out using validated age-appropriate instruments. For childhood and adolescence, QoL was measured using the KIDSCREEN-52 (29), a generic instrument measuring 10 dimensions of health-related QoL, while young adults with CP completed the WHOQOL-Bref questionnaire (30) with 24 items measuring their QoL in four areas. Both instruments cover three comparable domains of QoL, namely, physical well-being, psychological well-being, and social relationships. As one item used to measure physical well-being with the KIDSCREEN instrument was adapted for persons with CP in the version used in children and adolescents (14), this paper focused on the psychological well-being and social relationships domains. In both domains, the two instruments have a comparable number of items and response modalities; and a raw QoL score of 0–100 is obtained, with a higher score indicating a higher QoL (Supplementary Material 2).

In all waves of the SPARCLE cohort, we collected information on participants' walking ability (GMFCS), fine motor skills [Bimanual Fine Motor Function (BFMF)] (31), frequency of pain in the previous week, and seizures in the year predating the interview. At inclusion, intellectual ability was assessed either with formal IQ testing or using an algorithm based on a set of questions to child's parents (32) and thereafter categorized as dichotomous variable (<70/≥70), while the subtype of CP was available from the registers.

### Statistical Analyses

Of the SPARCLE3 participants (*n* = 176), 12 individuals who were out of the targeted age range at the time of interview (<22 or >27 years old) were excluded *a posteriori* from analysis.

Each of the two QoL domains was analyzed separately. We first described the distribution of QoL reported in childhood, adolescence, and young adulthood. To estimate the mean variation of raw QoL scores by age from childhood to young adulthood, we used a generalized linear mixed-effect model with random intercept (SAS 9.4, SAS Institute Inc., Cary, NC, USA) adjusted for parental education level at inclusion in order to limit non-response effects. All models were also adjusted for sex and region. In order to identify factors potentially associated with QoL variation from childhood to young adulthood, we added separately to the previous model the following variables: pain frequency in the previous week, seizures in the previous year, GMFCS, BFMF (all measured in each wave of follow-up), and IQ and CP subtypes (measured at inclusion). We also tested for interaction with age. A multivariate model that controlled for significant variables and interaction with age (*p* < 0.20) was performed. A descending step-by-step method was applied in order to reduce this model. The criterion for statistical significance was *p* < 0.05. To evaluate the impact of type of reporting on our results, we performed sensitivity analyses by excluding young adults with CP who were unable to self-respond to the questionnaire.

Then, in order to identify potential different trajectories of QoL variation from childhood to adulthood, we used a shape-based clustering method (kmlShape, R package v0.9.5, Genolini, 2016) that uses Fréchet means and Fréchet distances. This method uses a set of several parametric and non-parametric criteria [such as Calinski–Harabasz, Ray–Turi, Davies–Bouldin, Bayesian information criterion (BIC), and Akaike information criterion (AIC)] to determine the correct number of clusters (33). Individuals with CP who participated in all waves of the SPARCLE cohort were selected. Due to the small number of individuals in each identified cluster, we performed only descriptive analysis to determine whether profiles of individuals with CP identified in these clusters of QoL trajectories may be defined by impairment characteristics in childhood, adolescence, and young adulthood. We therefore considered pain frequency in the previous week, seizures in the previous year, GMFCS, BFMF, IQ (childhood only), and CP subtype (childhood only).

## Results

Our longitudinal sample included 164 young adults with CP (40.8% of eligible subjects) of whom more than two-thirds self-reported (with or without assistance) to the SPARCLE 3 questionnaire (*n* = 111, 67.7%). A total of 130 subjects participated in all the waves of the SPARCLE cohort with a similar self-report rate (*n* = 89, 68.5%).

### Sociodemographic and Impairment Characteristics

Young adults assessed in our SPARCLE3 longitudinal sample had a mean age of 24.3 years [standard deviation (SD) ± 1.6 years] at the time of interview, with a male-to-female ratio of 1.2. A large majority (77.4%) lived in urban or semi-urban areas (population ≥3,000 inhabitants), less than a third (29.3%) lived independently (alone, with a partner, or in cohabitation), and less than half (44.5%) were employed or in education when interviewed. Individuals who participated in all waves of the SPARCLE cohort had similar sociodemographic characteristics (Table 1).

**Table 1:** Sociodemographic characteristics of young adults with CP: SPARCLE cohort—France, Germany, Italy, and Sweden.

	**All participants (*****n*** **=** **164)**		**Participants in all waves (*****n*** **=** **130)**	
	***N***	**%**	***n***	**%**
**Region**				
Southeast France	37	22.6	20	15.4
Southwest France	29	17.7	22	16.9
Northwest Germany	49	29.9	42	32.3
Central Italy	20	12.2	17	13.1
Western Sweden	29	17.7	29	22.3
**Sex**				
Male	90	54.9	72	55.4
Female	74	45.1	58	44.6
**Age (years)**				
22	31	18.9	24	18.5
23	21	12.8	11	8.5
24	37	22.6	34	26.1
25	37	22.6	33	25.4
26	20	12.2	15	11.5
27	18	10.9	13	10.0
Means (SD)	24.3	(1.6)	24.3	(1.5)
**Parental education level**				
Did not complete secondary education	40	24.4	30	23.1
Secondary education	99	60.4	79	60.8
Tertiary education	25	15.2	21	16.1
**Size of unit of residence (inhabitants)**				
<3,000	36	22.0	31	23.8
3,000–200,000	63	38.4	49	37.7
>200,000	64	39.0	49	37.7
Missing	1	0.6	1	0.8
**Lifestyle**				
Living with partner	12	7.3	11	8.5
Living alone	31	18.9	23	17.7
Living in cohabitation	5	3.1	5	3.9
Living with parents	90	54.9	73	56.1
In care facilities	23	14.0	16	12.3
Other	3	1.8	2	1.5
**Current activity**				
Paid work	48	29.3	38	29.2
Non-paid work	4	2.4	2	1.5
In education	21	12.8	18	13.9
Unemployed	17	10.4	14	10.8
Permanently sick or disabled	45	27.4	32	24.6
Other	29	17.7	26	20.0

*CP, cerebral palsy; SD, standard deviation*.

The CP subtypes were spastic 75.6%, dyskinetic 16.5%, and ataxic 7.3%. At inclusion, 32.3% of participants had severe gross motor function limitations (GMFCS IV–V), 24.4% had severe BFMF impairment (BFMF IV–V), 51.8% had intellectual impairment (IQ <70), and 18.3% had seizures in the previous year. Level of impairment and associated conditions remained stable over time. More than two episodes of pain in the week prior to inclusion were reported by 32.3% of participants. No between-region heterogeneity was found for any of these variables (Table 2).

**Table 2:** Impairment characteristics of young adults with CP: SPARCLE cohort—France, Germany, Italy, and Sweden.

	**All participants**								**Participants in all waves**					
	Inclusion^*^ **(*****n*** **=** **164)**		**Childhood (*****n*** **=** **147)**		**Adolescence (*****n*** **=** **147)**		**Adulthood (*****n*** **=** **164)**		**Childhood (*****n*** **=** **130)**		**Adolescence (*****n*** **=** **130)**		**Adulthood (*****n*** **=** **130)**	
	***n***	**%**	***n***	**%**	***n***	**%**	***n***	**%**	***n***	**%**	***n***	**%**	***n***	**%**
**GMFCS**														
I–II	81	49.4	71	48.3	75	51.0	82	50.0	64	49.2	65	50.0	67	51.5
III	30	18.3	29	19.7	20	13.6	20	12.2	23	17.7	19	14.6	16	12.3
IV–V	53	32.3	47	32.0	52	35.4	62	37.8	43	33.1	46	35.4	47	36.2
**Bimanual Fine Motor Function**														
I. Without restriction//limitation	49	29.9	40	27.2	47	32.0	50	30.5	38	29.2	38	29.2	41	31.5
II–III. Moderate restrictions	75	45.7	70	47.6	70	47.6	68	41.5	60	56.2	65	50.0	53	40.8
IV–V. Severe restrictions	40	24.4	37	25.2	30	20.4	46	28.0	32	24.6	27	20.8	36	27.7
Intellectual impairment^*^														
≥70	77	47.5	-	-	-	-	-	-	66	51.2	-	-	-	-
<70	85	52.5	-	-	-	-	-	-	63	48.8	-	-	-	-
Missing	2		-	-	-	-	-	-	1		-	-	-	-
**Seizures in the previous year**														
No seizures (with or without medication)	134	81.7	119	80.9	117	79.6	135	82.3	106	81.5	102	78.5	110	84.6
Seizures	30	18.3	28	19.1	30	20.4	29	17.7	24	18.5	28	21.5	20	15.4
**Frequency of pain in previous week**														
None	67	41.4	60	41.4	45	31.0	46	28.2	53	41.1	38	29.5	36	27.9
Once or twice	42	25.9	38	26.2	33	22.8	35	21.5	31	24.0	29	22.5	27	20.9
Frequent	53	32.7	47	32.4	68	46.9	82	50.3	45	34.9	62	48.0	66	51.2
Missing	2		2		1		1		1		1		1	
Cerebral palsy subtype^*^														
Unilateral spastic	51	31.3	-	-	-	-	-	-	41	31.5	-	-	-	-
Bilateral spastic	73	44.8	-	-	-	-	-	-	59	45.4	-	-	-	-
Dyskinetic	27	16.6	-	-	-	-	-	-	23	17.7	-	-	-	-
Ataxic	12	7.4	-	-	-	-	-	-	7	5.4	-	-	-	-
Missing	1		-	-	-	-	-	-	0		-	-	-	-

*CP, cerebral palsy; GMFCS, Gross Motor Function Classification System*.

* *Data provided at inclusion in the SPARCLE cohort in childhood (n = 147) or in adolescence (n = 17)*.

### Quality of Life

**Figure 2:**
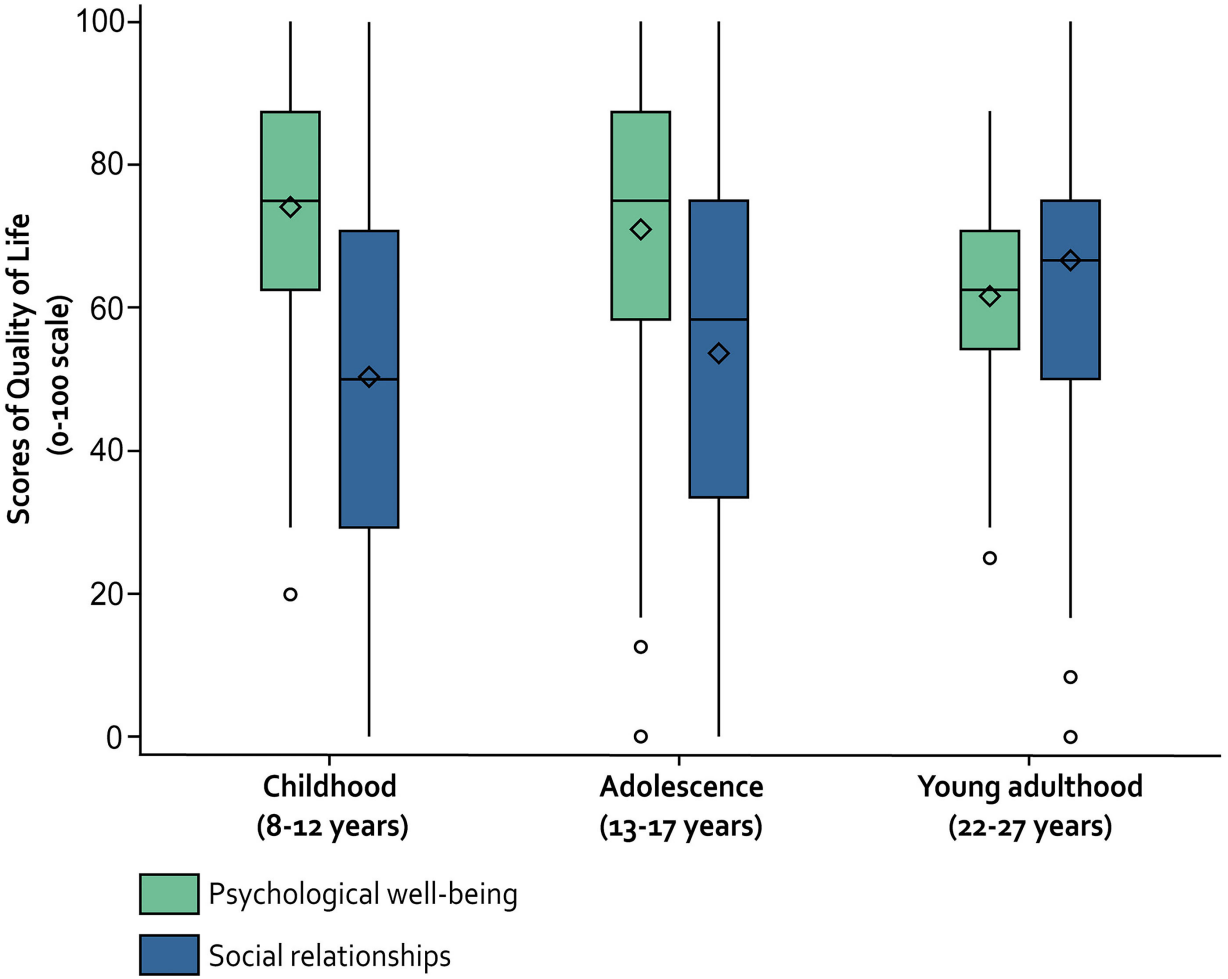
Distribution of QoL scores of individuals with CP from childhood to young adulthood—SPARCLE cohort—France, Germany, Italy, Sweden.

Table 3 and Figure 2 present the distribution of QoL reported in childhood (T1), adolescence (T2), and young adulthood (T3) for the psychological well-being and social relationships domains. Median scores were >60 for each domain in young adulthood, with a wider variation for social relationships. From childhood to young adulthood, psychological well-being showed a significant linear decrease by an average of 0.78 points per year (95% CI −0.99 to −0.56), whereas QoL in the social relationships domain showed a significant linear increase by an average of 1.24 points per year (95% CI 0.92 to 1.55; models adjusted for region, sex, and parental level of education). Similar variations were found in the group of individuals who participated in all waves (Table 3).

**Table 3:** Distribution and variation of QoL of individuals with CP from childhood to young adulthood: SPARCLE cohort—France, Germany, Italy, and Sweden.

	**All participants (*****n*** **=** **164)**		**Participants in all waves (*****n*** **=** **130)**	
	**Psychological well-being**	**Social relationships**	**Psychological well-being**	**Social relationships**
**Childhood**				
*n*	142	140	123	119
Mean (SD)	73.9 (18.1)	50.2 (26.7)	74.2 (18.6)	51.3 (26.2)
[Min–max]	[20.0–100.0]	[0.0–100.0]	[20.0–100.0]	[0.0–100.0]
**Adolescence**				
*n*	144	143	123	119
Mean (SD)	71.1 (20.7)	53.5 (27.5)	73.5 (19.2)	56.7 (26.7)
[Min–max]	[0.0–100.0]	[0.0–100.0]	[16.7–100.0]	[0.0–100.0]
**Young adulthood**				
*n*	155	160	123	119
Mean (SD)	61.7 (13.2)	66.7 (20.8)	63.1 (12.5)	68.3 (19.5)
[Min–max]	[25.0–87.5]	[0.0–100.0]	[25.0–87.5]	[8.3–100.0]
Change in QoL scores^*^				
β	−0.78	1.24	−0.81	1.12
[95% CI]	[−0.99 to −0.56]^†^	[0.92 to 1.55]^†^	[−1.04 to −0.58]^†^	[0.79 to 1.46]^†^

*QoL, quality of life; CP, cerebral palsy*.

* *β coefficients and 95% CI estimated by generalized linear mixed-effect model with random intercept adjusted for region, sex, and parental education level. β coefficients show the average difference in quality of life by 1 year of age*.

† *95% CI excluding zero*.

The β coefficients in Table 4 show average variations in QoL by impairment severity, seizure problems, and pain. Whatever their age, individuals with the more severe phenotypes of CP reported, on average, a lower QoL for psychological well-being and, more importantly, for social relationships compared with those less severely impaired. The severity of motor impairment significantly affected QoL in social relationships but had no significant effect on psychological well-being. On the contrary, IQ and seizures had a significant impact in both domains. In the final multivariate models, individuals with the most severe BFMF limitation (groups II–III and IV–V) reported significantly impaired QoL as compared with those without limitation (BFMF I) in the social relationships domain of 5.85 points (95% CI −11.08 to −0.63) and 8.49 points (95% CI −15.03 to −1.96), respectively. Similarly, seizures were significantly associated with lower QoL in the social relationships domain (β −10.60, 95% CI −16.61 to −4.48). Individuals with an IQ <70 had a lower QoL in psychological well-being (β −4.33, 95% CI −8.06 to −0.60) and in social relationships (β −9.44, 95% CI −14.74 to −4.14). Frequent pain was associated with a lower QoL only in psychological well-being (β −6.27, 95% CI −9.71 to −2.83). The associated factors identified in the multivariate models explained around 16 and 18% (*R*^2^) of the variation of QoL in the psychological well-being and social relationships domains, respectively.

**Table 4:** Average variations (by 1 year of age) in QoL by impairment severity, pain, and seizure problems in individuals with CP: SPARCLE cohort—France, Germany, Italy, and Sweden.

	**Psychological well-being**		**Social relationships**	
	**β^a^**	**95% CI^b^**	**β^a^**	**95% CI^b^**
Models considering each impairment separately^*^				
**GMFCS**				
I–II	0.00	Ref.	0.00	Ref.
III	−2.50	[−7.52 to 2.52]	−3.74	[−10.89 to 3.42]
IV–V	−2.06	[−6.14 to 2.03]	−7.72	[−13.50 to −1.93]^†^
**BFMF**				
I	0.00	Ref.	0.00	Ref.
II–III	−0.65	[−4.39 to 3.09]	−7.99	[−13.34 to −2.65]^†^
IV–V	−4.28	[−8.94 to 0.39]	−12.42	[−18.94 to −5.89]^†^
**IQ**				
≥70	0.00	Ref.	0.00	Ref.
<70	−3.99	[−7.85 to −0.13]^†^	−13.50	[−18.70 to −8.30]^†^
**Seizure in the previous year**				
No (with or without medication)	0.00	Ref.	0.00	Ref.
Seizures	−5.22	[−9.47 to −0.98]^†^	−14.16	[−20.11 to −8.21]^†^
**Frequency of pain in the previous week**				
None	0.00	Ref.	0.00	Ref.
Once or twice	−1.44	[−5.25 to 2.37]	0.59	[−5.01 to 6.18]
Frequent	−6.08	[−9.52 to −2.63]^†^	−4.19	[−9.28 to 0.91]
Multivariate model^*^				
**BFMF**				
I			0.00	Ref.
II–III			−5.85	[−11.08 to −0.63]^†^
IV–V			−8.49	[−15.03 to −1.96]^†^
**IQ**				
≥70	0.00	Ref.	0.00	Ref.
<70	−4.33	[−8.06 to −0.60]^†^	−9.44	[−14.74 to −4.14]^†^
**Seizure in the previous year**				
No (with or without medication)			0.00	Ref.
Seizures			−10.60	[−16.71 to −4.48]^†^
**Frequency of pain in the previous week**				
None	0.00	Ref.		
Once or twice	−1.28	[−5.09 to 2.53]		
Frequent	−6.27	[−9.71 to −2.83]^†^		
*R*^2^ (%)	15.85		17.95	

*BFMF, Bimanual Fine Motor Function; GMFCS, Gross Motor Function Classification System; IQ, intelligence quotient; QoL, quality of life; CP, cerebral palsy*.

* *All generalized linear mixed-effect model with random intercept models were adjusted for region, sex, and parental education level*.

a *β coefficients show the average difference in quality of life between the relevant category and the reference category (Ref.). β coefficients <0 indicate a lower quality of life in the relevant category compared with the reference category*.

b *CIs were calculated by bootstrapping*.

† *95% CI excluding zero*.

**Figure 3:**
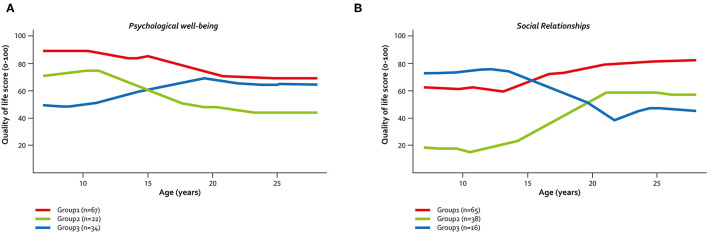
Identified QoL trajectories in individuals with CP. **(A)** QoL trajectories in psychological well-being. **(B)** QoL trajectories in social relationships—SPARCLE cohort—France, Germany, Italy, Sweden.

The clustering method revealed various shapes of QoL trajectories in both domains. For psychological well-being (Figure 3A), two groups of individuals showed a parallel trajectory, with a slight decrease of QoL during adolescence before stabilization in young adulthood. However, one group started with a high QoL in childhood (Group 1, *n* = 67 (54.5%), T1 QoL mean score (MS) = 87.1, T3-MS = 68.5), while the other had a moderate QoL in childhood (Group 2, *n* = 22 (17.9%), T1-MS = 68.6, T3-MS = 45.5). A third group, which comprised about one-quarter of the sample (*n* = 34, 27.6%), had a different profile, with low QoL during childhood, an increase during adolescence, and stabilization in young adulthood at a level close to that of Group 1 (T1-MS = 52.6; T3-MS = 64.0). Individuals who had moderate and low psychological well-being in childhood seemed to have more frequent seizures (22.7 and 20.6% in Groups 2 and 3, respectively) and pain (45.5 and 44.1% in Groups 2 and 3, respectively) in childhood than those in Group 1 with high initial QoL (10.4% had seizures and 23.9% had frequent pain). Individuals in Group 3 had a similar distribution of seizures and pain in adolescence as in childhood, whereas those in Group 2 reported more frequent seizures and pain in adolescence than in childhood. In Group 2 and Group 3, we observed a marked increase in the proportion of young adults who reported frequent pain, while seizures seemed to be less frequent than in adolescence (Table 5). Three clusters of trajectories were also identified for the social relationships domain (Figure 3B). The first two clusters showed an increase in QoL during adolescence that continued in a slight degree during young adulthood for Group 1 (*n* = 65, 54.6%) and became stable for Group 2 (*n* = 38, 32.8%). But these two groups had an entirely different profile in childhood, with moderate QoL in Group 1 (T1-MS = 63.8) and a very low QoL in Group 2 (T1-MS = 23.6). A third group (Group 3, *n* = 16, 13.5%) had a moderate QoL in childhood (T1-MS 66.1) that markedly decreased until early young adulthood (T3-MS = 46.9). Young adults in Group 2 had more severe phenotypes (higher proportions of GMFCS IV–V, BFMF III–V, IQ <70, and seizures) than those in Group 1. No differences were observed between Groups 1 and 3 for impairments in adolescence, while the proportion of individuals with frequent pain markedly increased in Group 3 and the proportion of individuals who experienced seizures decreased in Group 2 between adolescence and young adulthood (Table 5).

**Table 5:** Distribution of impairment severity, pain, and seizure problems from childhood to young adulthood by identified QoL trajectories in individuals with CP: SPARCLE cohort—France, Germany, Italy, and Sweden.

	**Psychological well-being**						**Social relationships**					
	**Group 1**		**Group 2**		**Group 3**		**Group 1**		**Group 2**		**Group 3**	
	**(*****n*** **=** **67)**		**(*****n*** **=** **22)**		**(*****n*** **=** **34)**		**(*****n*** **=** **65)**		**(*****n*** **=** **38)**		**(*****n*** **=** **16)**	
	***n***	**%**	***n***	**%**	***n***	**%**	***n***	**%**	***n***	**%**	***n***	**%**
***Childhood***												
**GMFCS**												
I–II	32	47.8	14	63.6	15	44.1	37	56.9	15	39.5	8	50.0
III	12	17.9	3	13.6	7	20.6	12	18.5	7	18.4	2	12.5
IV–V	23	34.3	5	22.7	12	35.3	16	24.6	16	42.1	6	37.5
**BFMF**												
I	20	29.8	9	40.9	9	26.5	27	41.5	6	15.8	4	25.0
II–III	32	47.8	10	45.5	16	47.0	28	43.1	20	52.6	9	56.2
IV–V	15	22.4	3	13.6	9	26.5	10	15.4	12	31.6	3	18.8
IQ^*^												
≥70	35	52.2	10	45.4	20	58.8	44	67.7	12	31.6	8	50.0
<70	32	47.8	12	54.6	14	41.2	21	31.3	26	68.4	8	50.0
**Seizure in the previous year**												
No (with or without medication)	60	89.6	17	77.3	27	79.4	57	87.7	27	71.0	16	100.0
Seizures	7	10.4	5	22.7	7	20.6	8	12.3	11	29.0	0	0.0
**Frequency of pain in the previous week**												
None	35	53.0	7	31.8	10	29.4	24	37.5	21	55.3	7	43.8
Once or twice	15	22.7	5	22.7	9	26.5	16	25.0	7	18.4	6	37.5
Frequent	16	24.3	10	45.5	15	44.1	24	37.5	10	26.3	3	18.7
Missing	1		0		0		1		0		0	
CP subtype^*^												
Unilateral spastic	15	22.4	10	45.4	13	38.2	24	36.9	8	21.1	5	31.3
Bilateral spastic	34	50.7	8	36.4	15	44.1	31	47.7	15	39.5	9	56.2
Dyskinetic	13	19.4	2	9.1	6	17.7	7	10.8	11	28.9	2	12.5
Ataxic	5	7.5	2	9.1	0	0.0	3	4.6	4	10.5	0	0.0
***Adolescence***												
**GMFCS**												
I–II	31	46.3	14	63.6	18	52.9	37	56.9	17	44.7	8	50.0
III	12	17.9	1	4.6	4	11.8	11	16.9	4	10.5	2	12.5
IV–V	24	35.8	7	31.8	12	35.3	17	26.2	17	44.7	6	37.5
**BFMF**												
I	20	29.9	7	31.8	11	32.4	26	40	6	15.8	5	31.2
II–III	36	53.7	13	59.1	13	38.2	32	49.2	20	52.6	8	50.0
IV–V	11	16.4	2	9.1	10	29.4	7	10.8	12	31.6	3	18.8
**Seizures in the previous year**												
No (with or without medication)	60	89.5	14	63.6	26	76.5	57	87.7	25	65.8	14	87.5
Seizures	7	10.5	8	36.4	8	23.5	8	12.3	13	34.2	2	12.5
**Frequency of pain in the previous week**												
None	23	34.3	3	13.6	11	32.3	21	32.3	11	28.9	5	31.2
Once or twice	17	25.4	6	27.3	6	17.7	11	16.9	11	28.9	5	31.2
Frequent	27	40.3	13	59.1	17	50.0	33	50.8	16	42.2	6	37.5
***Young adulthood***												
**GMFCS**												
I–II	35	52.2	12	54.5	17	50.0	40	61.5	17	44.7	7	43.8
III	10	14.9	2	9.1	3	8.8	8	12.3	4	10.6	3	18.7
IV–V	22	32.9	8	36.4	14	41.2	17	26.2	17	44.7	6	37.5
**BFMF**												
I	21	31.3	7	31.8	13	38.2	29	44.6	7	18.4	4	25.0
II–III	28	41.8	10	45.5	11	32.4	26	40.0	16	42.1	8	50.0
IV–V	18	26.9	5	22.7	10	29.4	10	15.4	15	39.5	4	25.0
**Seizures in the previous year**												
No (with or without medication)	62	92.5	16	72.7	29	85.3	63	96.9	26	68.4	14	87.5
Seizures	5	7.5	6	27.3	5	14.7	2	3.1	12	31.6	2	12.5
**Frequency of pain in the previous week**												
None	27	40.3	2	9.1	5	14.7	16	24.6	15	39.5	2	12.5
Once or twice	16	23.9	5	22.7	5	14.7	17	26.2	5	13.1	2	12.5
Frequent	24	35.8	15	68.2	24	70.6	32	49.2	18	47.4	12	75.0

*BFMF, Bimanual Fine Motor Function; GMFCS, Gross Motor Function Classification System; IQ, intelligence quotient; CP, cerebral palsy*.

* *Data only provided in childhood*.

In our sensitivity analyses, long-term trajectories did not change after excluding individuals with proxy responses. The associations between frequency of pain and psychological well-being and between seizures and social relationships were still observed. Conversely, there was no longer an association between BFMF and social relationships, whereas intellectual disability was no longer associated with lower QoL in either domain.

## Discussion

In this longitudinal analysis of the SPARCLE cohort, we observed that QoL in individuals with CP linearly decreased from childhood to young adulthood in the domain of psychological well-being, whereas it linearly increased in social relationships. Severity of impairment was associated with reduced QoL in all life phases studied (childhood, adolescence, and young adulthood): motor impairment with social relationships, and intellectual impairment with psychological well-being and social relationships. At all time periods, frequent pain reduced psychological well-being, and seizures were associated with poorer QoL in the social relationships domain. Using clustering methods, we identified a group of young adults with CP who presented a reverse pattern to the overall trajectory of QoL in both domains.

To our knowledge, our study is the first to investigate long-term trajectories of QoL in young people with CP from childhood to early adulthood using individual longitudinal data. A Dutch study by Tal et al. also investigated long-term trajectories of QoL dimensions related to psychological and social functioning but reconstituted a longitudinal sequence with four neighboring 4-year follow-up cohorts: toddlers (*n* = 97), children (*n* = 116), adolescents (*n* = 108), and young adults (*n* = 103) (21). Unlike our findings, QoL in the domains of psychological and social functioning did not change significantly over time. Apart from study design, a series of differences between the two studies may explain the divergent results. Whereas, Tal et al. considered aspects of life directly influenced by disability, health problems, or treatments (4, 5), we used a broader definition of subjective QoL that encompassed the concept of holistic well-being (6). Although QoL and HRQoL reflect the individual's subjective perception of their position in life (30), these outcomes are substantially different (34, 35). In addition, the sample used by Tan et al., consisting of individuals with CP recruited from rehabilitation centers, had less severe impairment than those in our study and did not include young adults with intellectual disabilities. However, our results relating to long-term trajectories of QoL were unchanged when we excluded from analysis young adults with intellectual disabilities or those with proxy reports (94% with intellectual disability, 60% with GMFCS IV–V). Interestingly, we demonstrated that change in QoL with age was not one-directional. Our clustering analysis showed that 26.2 and 12.3% of the study population displayed a reverse trajectory of QoL in the psychological well-being and social relationships domains, respectively, as compared with their peers. Inclusion in these groups did not appear to be related to a specific profile of disability severity. The proportion of proxy reports did not significantly differ between these particular groups and the other two groups. Nevertheless, because of the small size of the groups, we were not able to investigate whether these reverse QoL trajectories were determined by specific factors. In young adults with CP, the association between severity of motor impairment and QoL appears to depend on the domain considered. Three cross-sectional studies investigated the association between GMFCS levels and psychological well-being (17, 36, 37) and reported conflicting results. Although they all used the concept of HRQoL, two studies showed no significant difference in perceived mental health across the range of GMFCS levels (36, 37), in agreement with our findings, whereas one study (17) showed that young adults with the most severe gross motor function impairments (GMFCS III–V) were more likely to have better perceived mental health than those less severely impaired (GMFCS I–II). On the other hand, our findings suggesting that severe motor impairment may affect the subjective perception of social relationships during childhood, adolescence, and young adulthood are not consistent with the findings of Tan et al. (21). While analysis of the SPARCLE data collected in childhood and adolescence identified GMFCS as a factor associated with QoL in the social relationships domain (14), it is interesting to note that fine motor function appears to have a more determining role for social interaction in young adults with CP. However, this association disappeared when individuals who proxy-reported their QoL were excluded (sensitivity analyses). The interpretation of these latter results is difficult. First, they may point to a specific social condition of individuals with the most severe profiles. Second, we cannot exclude that our findings may be supported by under- or overestimation of QoL in the social relationships domain due to proxy responses. Individuals reporting on behalf of another person often have a substantially different perspective on the QoL domain under consideration (23). To our knowledge, identification of intellectual impairment and seizures as factors associated with lower QoL in the social relationships domain from childhood to young adulthood has never previously been reported in the literature. Interestingly, these associated factors have previously been identified as determinants of non-inclusion at school (38), whereas severe intellectual impairment and seizures have also been reported as factors associated with unemployment (39–42). Because a recent qualitative study has highlighted the relationship between social participation and QoL (43), it is possible that the factors associated with lower QoL in the social relationships domain identified in this study indirectly reflect limited access to education or employment or, more generally, restrictions on social participation. Given that pain has been shown to be strongly associated with reduced psychological QoL in childhood and adolescence (12–14), it was not surprising to observe that this association continued in young adulthood. This finding confirms the importance of addressing pain across the life span in people with CP, especially as the prevalence of frequent pain is high regardless of age (44–49). In addition, fatigue frequently co-occurring with pain (48, 50) should be considered in further analyses of the role of pain. Our models for QoL in the psychological well-being and social relationships domains explained 16 and 18%, respectively, of the variance in outcome, which may seem low. However, this is not surprising because from childhood to early adulthood, people with CP face numerous and complex barriers that can impact QoL, and these barriers are not limited to the severity of impairment and the presence and frequency of pain (51–53). The SPARCLE cohort is, to the best of our knowledge, the largest prospective study of people with CP from childhood to young adulthood. Our sample was selected from European population-based registries or various sources in northwest Germany, thus limiting selection bias. We purposely overrepresented the most severe forms of CP to investigate this less common group in depth. However, our choice calls into question the external validity of our findings, notably for QoL trajectories. Moreover, we cannot exclude that dropouts during follow-up led to the exclusion of the most severe cases. All analyses were adjusted for the predictors of non-response identified in this study to limit the impact of dropout. Despite these potential differential errors, it is interesting to note that the participants in the SPARCLE cohort who contributed to the third wave had a similar distribution of impairment and seizures when they were children as the individuals born between 1990 and 2006 and recorded by the SCPE (54).

No instrument has been developed to adequately measure QoL over such a wide age range. Therefore, we used two different instruments based on the same concept and the WHO definition (7). As mentioned earlier, these two instruments presented the advantage of having a comparable number of items and response modalities, and of providing QoL scores on a 0–100 scale. Nevertheless, we cannot be sure that this choice is valid for bridging the age gap between adolescence and young adulthood. First, it is likely that the two instruments have different Rasch scaling. Therefore, it would have been better to use transformed Rasch scores that take into account discriminant ability of items rather than raw scores as we did. However, we were limited by the lack of data on the psychometric properties and population norms of the WHOQOL-Bref in our specific population of European young adults, whereas Rasch scores exist for the KIDSCREEN-52 and were constructed to meet the population norms of European children and adolescents (mean = 50, SD = 10) (29). We considered additional analyses to explore this point, but none seemed satisfactory. Among them, use of the KIDSCREEN index, a short form of the KIDSCREEN-52, which was collected in all waves of this study, was considered; but unfortunately, this instrument only measures overall QoL and not domain-specific QoL. The exact opposite is true for the WHOQOL-Bref. This limited our ability to use the KIDSCREEN index as a validation tool in our study. Finally, we performed the analyses by pooling self-reports and proxy reports to describe QoL across the whole range of severity of our population, considering that regardless of the respondent, the report was the best available estimate of QoL. We cannot rule out a potential for under- or overestimation of QoL when using proxy reports (23). But the majority of our findings did not change when proxy reports were excluded from our sensitivity analyses, reducing the impact of this limitation. Only the association between BFMF and QoL in the social relationships domain was no longer significant, which may nuance our findings in this particular domain. Furthermore, given that the majority of individuals with intellectual disability (IQ <70) was in the proxy-reporting group (*n* = 50/85, 58.9%) while almost all of those with an IQ ≥ 70 were able to answer the questionnaire themselves (*n* = 75/77, 97.4%), the significant impact of intellectual disability on QoL trajectories observed in the total sample was no longer significant when the analyses were restricted to self-reports.

## Conclusion

Identification of QoL trajectories and their associated factors improves our knowledge of the experience of individuals with CP up till young adulthood. Further studies are needed to better understand which of the disability, personal, and contextual factors have the most influence on the differently shaped long-term trajectories of QoL.

## Data Availability Statement

The data analyzed in this study is subject to the following licenses/restrictions: dataset is accessible after submission of a scientific project and approval of the project by the investigators of each participating country. Requests to access these datasets should be directed to Catherine Arnaud, catherine.arnaud@univ-tlse3.fr.

## Ethics Statement

The relevant Ethics and Regulatory authorizations were sought in each country and the study fully approved by the below: France: The data were collected and stored in accordance with the reference methodology MR003 [Declaration No. 2205849 at the Commission for Data Protection and Liberties (CNIL)] each patient having been informed individually of the research under Article L1122-1 of the Public Health Code. Germany: Ethikkommission der Universität zu Lübeck [AZ 18-172]. Italy: Comitato Etico Lazio 1c/o A.O. San Camillo Forlanini [2143/CE Lazio 1]. Sweden: Regional Ethical Review Board in Göteborg. All young people with CP or their legal representatives gave written informed consent to participate, or non-opposition where appropriate.

## Author Contributions

NVEB and CA conceived and designed the analysis. NV performed the analysis under the supervision of VE and drafted the manuscript. CA, JF, SS-S, UT, KH, and MM conceived and designed the cohort and contributed to the longitudinal data. CA, CD, SS-S, UT, KH, and MM designed the third wave of follow-up, which was managed by CD. All authors contributed to the article, provided critical feedback, and approved the submitted version.

## Funding

This work was supported by the Deutsche Forschungsgemeinschaft (DFG) and the French Agence Nationale de la Recherche (ANR) (DFG-ANR N°316684170) in Germany and France; the Sunnerdahls Handikappfond, the Swedish state under the agreement between Swedish government and the country councils, the ALF-agreement (ALFGBG-726001) in Sweden; and the Fondazione Carivit in Italy. The funders were not involved in any way in the preparation of this manuscript or the decision to submit it.

## Conflict of Interest

The authors declare that the research was conducted in the absence of any commercial or financial relationships that could be construed as a potential conflict of interest.

## Publisher's Note

All claims expressed in this article are solely those of the authors and do not necessarily represent those of their affiliated organizations, or those of the publisher, the editors and the reviewers. Any product that may be evaluated in this article, or claim that may be made by its manufacturer, is not guaranteed or endorsed by the publisher.

## Abbreviations

BFMF: Bimanual Fine Motor Function
CI: confidence interval
CP: cerebral palsy
GMFCS: Gross Motor Function Classification System
HRQoL: health-related quality of life
IQ: intelligence quotient
MS: mean score
OR: odds ratio
QoL: quality of life
SCPE: Surveillance of Cerebral Palsy in Europe network
SPARCLE: Study of PARticipation of children with Cerebral palsy Living in Europe
WHO: World Health Organization.
