# Supplementary material for: Quality of Life in Young Adults With Cerebral Palsy: A Longitudinal Analysis of the SPARCLE Study
Source: Front Neurol. 2021 Nov 1;12:733978. doi: 10.3389/fneur.2021.733978 (PMC8591289; doi:10.3389/fneur.2021.733978)
Supplement: Supplementary file 1 [file Table_1.docx]

**Supplementary material 1. Familial and impairments characteristics at inclusion and participation in the third wave of follow-up – SPARCLE cohort – France, Germany, Italy, Sweden**

|  |  | **SPARCLE 3 non-participants (n=226)** | |  | **SPARCLE 3 participants (n=176)** | | **OR** | **95% CI** | **p** |
| --- | --- | --- | --- | --- | --- | --- | --- | --- | --- |
|  |  | **n** | **%** |  | **n** | **%** |  |  |  |
| **Models considering each variable separately*** | |  |  |  |  |  |  |  |  |
| **GMFCS** | |  |  |  |  |  |  |  | 0.3557 |
|  | I-II | 111 | 49.1 |  | 91 | 51.7 |  |  |  |
|  | III | 29 | 12.8 |  | 31 | 17.6 |  |  |  |
|  | IV-V | 86 | 38.1 |  | 54 | 30.7 |  |  |  |
| **IQ** | |  |  |  |  |  |  |  | 0.1421 |
|  | ≥ 70 | 87 | 38.5 |  | 83 | 47.2 |  |  |  |
|  | < 70 | 136 | 60.2 |  | 91 | 51.7 |  |  |  |
|  | Missing | 3 | 1.3 |  | 2 | 1.1 |  |  |  |
| **Parental Stress Index** | |  |  |  |  |  |  |  | 0.1292 |
|  | < 70 (Q1) | 45 | 21.1 |  | 50 | 28.4 |  |  |  |
|  | 70-100 (Q1-Q3) | 122 | 57.3 |  | 78 | 44.3 |  |  |  |
|  | > 100 (Q3) | 46 | 21.6 |  | 42 | 23.9 |  |  |  |
|  | Missing | 13 |  |  | 6 |  |  |  |  |
| **Family structure** | |  |  |  |  |  |  |  | 0.6354 |
|  | Married, living with together | 166 | 73.4 |  | 128 | 72.7 |  |  |  |
|  | Unmarried, living together | 20 | 8.9 |  | 19 | 10.8 |  |  |  |
|  | Single or separated living with parents | 9 | 4.0 |  | 4 | 2.3 |  |  |  |
|  | Single, living alone | 31 | 13.7 |  | 24 | 13.6 |  |  |  |
|  | Other | 0 | 0.0 |  | 1 | 0.6 |  |  |  |
| **Parental education level** | |  |  |  |  |  |  |  | 0.0005 |
|  | Tertiary education completed | 19 | 8.4 |  | 27 | 15.3 |  |  |  |
|  | Secondary education completed | 122 | 54.0 |  | 105 | 59.7 |  |  |  |
|  | Did not complete secondary education | 85 | 37.6 |  | 44 | 25.0 |  |  |  |
| **Final model*** | |  |  |  |  |  |  |  |  |
| **Parental education level** | |  |  |  |  |  |  |  | 0.0005 |
|  | Tertiary education completed | 19 | 8.4 |  | 27 | 15.3 | 1.0 | Ref. |  |
|  | Secondary education completed | 122 | 54.0 |  | 105 | 59.7 | 1.9 | [0.9-3.7] |  |
|  | Did not complete secondary education | 85 | 37.6 |  | 44 | 25.0 | 4.0 | [1.9-8.4] |  |

* Unconditional logistic regression models adjusted for age and sex. A multivariate model that controlled for significant variables and interaction with age (p<0.20) was performed. A descending step-by-step method was applied in order to reduce this model. The criterion for statistical significance was p<0.05.

GMFCS: Gross Motor Function Classification System; IQ: Intellectual Quotient; OR: Odd Ratio; CI: Confidence Interval.
