# Supplementary material for: Quality of Life in Young Adults With Cerebral Palsy: A Longitudinal Analysis of the SPARCLE Study
Source: Front Neurol. 2021 Nov 1;12:733978. doi: 10.3389/fneur.2021.733978 (PMC8591289; doi:10.3389/fneur.2021.733978)
Supplement: Supplementary file 2 [file Table_2.DOCX]

**Supplementary material 2. KIDSCREEN-52 and WHOQOL-Bref items used to estimate the scores of quality of life in Psychological well-being and Social relationships domains.**

| **Subdomains** | **KIDSCREEN-52 items** | **WHOQOL-Bref items** |
| --- | --- | --- |
| **Psychological well-being**  **(6 items)** | Has your life been enjoyable? | How much do you enjoy life? |
|  | Have you felt satisfied with your life? | To what extent do you feel your life to be meaningful? |
|  | Have you felt cheerful? | How well are you able to concentrate? |
|  | Have you had fun? | Are you able to accept your bodily appearance? |
|  | Have you felt pleased that you are alive? | How satisfied are you with yourself? |
|  | Have you been in a good mood? | How often do you have negative feelings such as blue mood, despair, anxiety, depression? |
| **Social relationships**  **(3 items)** | Have you been afraid of other girls and boys? | How satisfied are you with your personal relationships? |
|  | Have other girls and boys made fun with you? | How satisfied are you with your sex life? |
|  | Have other girls and boys bullied you? | How satisfied are you with the support you get from your friends? |
